# Prevalence of depression and anxiety among cancer patients

**Published:** 2014

**Authors:** Novin Nikbakhsh, Sussan Moudi, Setareh Abbasian, Soraya Khafri

**Affiliations:** 1 Department of Surgery, Babol University of Medical Sciences, Babol, Iran.; 2 Department of Psychiatry Babol University of Medical Sciences, Babol, Iran.; 3 Babol University of Medical Sciences, Babol, Iran.; 4 Department of Social Medicine and Health, Babol University of Medical Sciences, Babol, Iran.

**Keywords:** Cancer, Anxiety, Depression, Treatment

## Abstract

***Background: ***Depression and anxiety had negative effects on the quality of life of cancer patients, thus hospital anxiety and depression scale (HADS) is a useful instrument for screening these problems. This research was performed to assess the prevalence of their anxiety and depression.

***Methods:*** From 2012-2013, one hundred fifty patients with recent diagnosis of different cancers in Babol, Iran were assessed. A presumptive diagnosis of anxiety and depression was based on a four point 14-item HADS. The score of 0-7 means without clinical symptoms of anxiety or depression, 8-10 mild and 11-21 symptomatic anxiety or depression. The data were collected and analyzed.

***Results:*** Forty-four (29.3%) patients had mild anxiety, 25 (16.7%) symptomatic anxiety but mild and symptomatic depression were seen in 40 (26.7%) and 32 (21.3%) patients, respectively. There were significant relationships between anxiety, depression and the age group of the patients with higher frequency in older ages. There were significant relationships between anxiety and depression with the type of cancer and type of treatment. Breast and stomach cancer patients had the highest prevalence of anxiety and depression and the higher prevalence was observed in the patients who received chemotherapy as the single treatment.

***Conclusion: ***The results show that patients with breast and stomach cancer had the highest prevalence of anxiety and depression among all others cancer patients.

Depression and anxiety are not uncommon among people diagnosed with cancer. Stress is often a trigger for depression and anxiety, and cancer is one of the most stressful events that a person may experience. These conditions may interfere with cancer treatment. For example, the patients with untreated depression or anxiety may be less likely to take his cancer treatment medication and continue good health habits because of fatigue or lack of motivation. They may also withdraw from family or other social support systems, which means they will not ask for the needed emotional and financial support to cope with cancer. This in turn may result in increasing stress and feelings of despair (1). Routine screening for distress is internationally recommended as a necessary standard for good cancer care (2). Hospital anxiety and depression scale (HADS) is a useful instrument for screening depression and anxiety in clinical settings. It was developed by Zigmond and Snaith in 1983. Its purpose is to provide clinicians with an acceptable, reliable, valid and easy to use practical tool for identifying and quantifying depression and anxiety (3).

Despite the importance of depression or anxiety screening in cancer patients, there is no previous study dealing with the prevalence of these psychological disorders among cancer patients in Babol, therefore this research was performed to assess depression and anxiety in these patients.

## Methods

From 2012 through 2013 patients with recent diagnosis of breast, colorectal, stomach, esophagus, lung or thyroid cancer scheduled for surgery, chemotherapy, radiotherapy or combination therapy and referred to three main hospitals of Babol in Iran were investigated in the study. The patients with past history of psychological disorders were excluded from the study. 

The sample size was calculated as 150 cases (based on the estimation of 30% anxiety or depression in cancer patients with α=0.05 and d=0.20). A presumptive diagnosis of anxiety and depression was based on a four point 14-item Hospital Anxiety and Depression Scale (HADS). HADS has two subscales for anxiety (seven items) and for depression (seven items) (3)^.^ For each item, the participants respondents were asked to indicate which of the 4 options (rated from 3 to 0; score range, 0-42) comes closest to describing how they have been feeling in the past week. The score of 0-7 means without clinical symptoms of anxiety or depression, 8-10 mild anxiety or depression and 11-21 symptomatic anxiety or depression. The spectrum of depression means cumulation of symptomatic plus mild depression and the spectrum of anxiety means cumulation of symptomatic plus mild anxiety. The data were collected and analyzed using SPSS Version 16. The outcomes were compared with socio-demographic and medical characteristics of patients with chi-square test. 

## Results

During the study, one hundred-fifty cases with a recent diagnosis of breast, colorectal, stomach, esophagus, lung or thyroid cancer have been included in the study. One hundred forty-six (97.3%) cases were married, 3(2%) cases single and 1 (0.7%) divorced. Seventy-eight (52%) cases were females and 72 (48%) were males. Regarding educational levels, below diploma, diploma, higher than diploma and illiterate were seen in 35.3%, 22%, 7.4% and 35.3% cases, respectively. The mean age of the patients was 59.04±14.34 (range of 22-88) years. Most of the patients [126 cases (84%)] were scheduled for surgery and the others for chemotherapy or combination therapies. 

One hundred forty-nine (99.3%) cases had no family history of depression. Eighty-one (54%) patients had no clinical symptoms of anxiety, 44 (29.3%) mild anxiety, 25 (16.7%) with symptomatic anxiety and these rates were seen in 78 (52%), 40 (26.7%), 32 (21.3%) for depression, respectively. There were significant relationships between anxiety, depression and the age group of the patients (P=0.004 and 0.007, respectively) with higher frequency in older ages. There were no significant relationships between anxiety and depression with sex, marital status and the educational levels of the patients (p>0.05). The distribution of anxiety and depression in different cancers and treatments are presented in tables 1, 2. There were significant relationships between anxiety and depression with the type of cancer (P=0.001 and 0.003, respectively) and type of treatment (p<0.05).

**Table 1:** Distribution of anxiety and depression in different cancers

**Type of ** **Cancer**	**No clinical symptoms of anxiety** **N (%)**	**No clinical symptoms of depression** **N (%)**	**mild anxiety** **N (%)**	**mild depression** **N (%)**	**symptomatic anxiety** **N (%)**	**symptomatic depression** **N (%)**	**Total** **N (%)**
breast	16 (19.8)	16 (20.5)	22 (50)	21 (52.5)	8 (32)	9 (28.1)	46 (30.7)
colorectal	18 (22.2)	18 (23.1)	5 (11.4)	3 (7.5)	1 (4)	3 (9.4)	24 (16)
stomach	25 (30.9)	21 (26.9)	7 (15.9)	10 (25.0)	8 (32)	9 (28.1)	40 (26.7)
esophagus	11 (13.6)	11 (14.1)	6 (13.6)	3 (7.5)	2 (8)	5 (15.6)	19 (12.7)
lung	2 (2.5)	2 (2.6)	2 (4.5)	2 (5.0)	5 (20)	5 (15.6)	9 (6)
thyroid	9 (11.1)	10 (12.8)	2 (4.5)	1 (2.5)	1 (4)	1 (3.1)	12 (8)
Total	81 (100)	78 (100)	44 (100)	40 (100)	25 (100)	32 (100)	150 (100)

**Table 2:** Distribution of Anxiety and Depression in Different Treatments of Cancer

**Type of Treatment**	**0-7** **(no clinical symptoms of A or D)** **N (%)**	**8-10** **(mild A or D)** **n (%)**	**11-21** **(symptomatic A or D)** **n (%)**	**Total** **n (%)**
surgery	٭A:72 (57.1)	A:38 (30.2)	A:16 (12.7)	126 (100)
	٭٭D:71 (56.3)	D:35 (27.8)	D:20 (15.9)	
Chemotherapy	A:0	A:3 (33.3)	A:6 (66.7)	9 (100)
	D:0	D:2 (22.2)	D:7 (77.8)	
combination therapy	A:9 (60.0)	A:3 (20.0)	A:3 (20.0)	15 (100)
	D:7 (46.7)	D:3 (20.0)	D:5 (33.3)	
Total	A:81 (54.0)	A:44.0 (29.3)	A:25 (16.7)	150 (100)
	D:78 (52.0)	D:40 (26.7)	D:32 (21.3)	

٭ A: Anxiety

٭٭ D: Depression

## Discussion

In this study, the spectrum of depression (symptomatic plus mild depression) was 48% and the spectrum of anxiety (symptomatic plus mild anxiety) was 46%. Breast and stomach cancer patients had the highest prevalence of anxiety and depression which had similarities and differences with other researches in Iran or other countries (4-17). In breast cancer patients, the importance of body image and the influence of mastectomy on it, self-image and its effect on sex drive, can justify the higher frequency of anxiety and depression in this group. In gastrointestinal tract cancer patients, the high frequency of anxiety and depression can be related to the changes due to the disease itself or the effect of different treatments on the patient^, ^s appearance. Fatigue, malaise, weight loss and surgical consequences like colostomy are the common causes of anxiety and depression in these patients (18). 

In our study, anxiety and depression had significant associations with the type of treatment, high frequency was observed in the patients who received chemotherapy as a single treatment (66.7% had symptomatic depression and 77.8% symptomatic anxiety). This high frequency of anxiety and depression can be related to end-stage and poor prognosis position of the patient which cause the selection of chemotherapy as the single route of treatment. Past research suggests that psychological responses to cancers vary by age (6, 10, 11, 12, 14). In our study, anxiety and depression had higher frequencies in older ages. Old age increases the duration of disease, high probability of cancer metastasis and more disability and these conditions increase anxiety and depression in older patients. The weakness of our research was the absence of clinical structural interview with patients, which caused the probable diagnosis of anxiety and depression according to HADS. If structural interview had been performed, definite diagnosis could have been made. In conclusion, continuous screening for anxiety and depression is recommended as a necessary approach for good cancer care; on the other hand, after the diagnosis of clinically important psychological disorders, proper treatment interventions must be performed to improve the quality of life in these patients. 

The patients with breast and stomach cancer had the highest prevalence of anxiety and depression among all others cancer patients.

## Notes

**Funding:** This research was supported by the Vice-Chancellery for Research of Babol University of Medical Sciences (No: 1241).
